# Three-dimensional transesophageal echocardiography of the atrial septal defects

**DOI:** 10.1186/1476-7120-6-38

**Published:** 2008-07-18

**Authors:** Francisco-Javier Roldán, Jesús Vargas-Barrón, Clara Vázquez-Antona, Luis Muñoz Castellanos, Julio Erdmenger-Orellana, Ángel Romero-Cárdenas, Marco-Antonio Martínez-Ríos

**Affiliations:** 1 Echocardiography department. Instituto Nacional de Cardiología "Ignacio Chávez", Mexico City, Mexico; 2 Embryology department. Instituto Nacional de Cardiología "Ignacio Chávez", Mexico City, Mexico; 3 Catheterization laboratory. Instituto Nacional de Cardiología "Ignacio Chávez", Mexico City, Mexico

## Abstract

Transesophageal echocardiography has advantages over transthoracic technique in defining morphology of atrial structures. Even though real time three-dimensional echocardiographic imaging is a reality, the off-line reconstruction technique usually allows to obtain higher spatial resolution images. The purpose of this study was to explore the accuracy of off-line three-dimensional transesophageal echocardiography in a spectrum of atrial septal defects by comparing them with representative anatomic specimens.

## Introduction

Transesophageal echocardiography (TEE) has assumed an important role in atrial septal defects (ASD) study [1], however, when only two-dimensional (2D) images are used for diagnosis, anatomical details can be ignored [2] with significant therapeutic consequences. With the development of fast acquisition for imaging reconstruction, dynamic 3D echocardiography has provided new imaging planes improving the understanding of cardiac anatomy and physiology [3].

Although live/real time three-dimensional (3D) transthoracic and TEE have been recently introduced to the echocardiography armamentarium [4,5], off-line technique allows the use of higher spatial resolution transducers for image acquisition improving anatomical details definition [6]. The emergence of new therapeutic options for ASDs demands accurate delineation of their morphology and anatomical relationships [7]. The maximal diameter of the defect and the dimensions of the septal rims are essential parameters for the selection of optimal cases for device closure. Our aim was to evaluate if 3D-TEE might help to improve anatomical analysis of ASDs.

## Methods

In the group of patients with diagnosis of ASD by transthoracic echocardiography (TTE) who underwent clinically indicated TEE, six were prospectively selected to acquire additional planes for 3D reconstruction. The first case had a residual shunt after Ostium Secundum (OS) percutaneous closure. In the second one, TEE was a routine procedure before attempting percutaneous closure of an OS ASD. In the other cases TEE was requested for further information of different types of ASDs detected by transthoracic echocardiography: an Ostium Primun (OP) ASD, a Venous Sinus (VS) type, a Common Atrium (CA) and another more OS ASD with total anomalous pulmonary venous connection to coronary sinus.

Imaging acquisition was carried out with a multiplane 5-MHz TEE transducer connected to commercially available equipment (system Sonos 5500, Philips Electronics, Koninklijke, N). Images were acquired with the rotational scanning method every 3° and synchronized with the R-wave of electrocardiographic lead II and with the respiration rate and depth. The TEE transducer was placed at the mid-esophageal level to obtain an optimal view of the atrial septum. Once the target structure was identified, the transducer was adjusted to place the region of interest as close to the centre of the scan as possible and a sweep test was obtained to ensure that the region of interest would be captured by the most centered scan lines. 2D images were stored in magnetic optical disks for off-line analysis using the commercially available Echo-Scan v4.0 system (Tom-Tec Gmb H, Munich, Germany). When the 3D reconstruction was completed, it was possible to determine completely the endocardial surface of the interatrial septum.

The 2D and 3D images were compared and the pathologic specimens were examined. Analysis included time of image acquisition, image quality, size and shape of the defects and its relation with adjacent structures such as embryological remnants. 3DTEE images were compared with analogous anatomic specimens selected from the Museum of Pathology at the "Instituto Nacional de Cardiologia", a collection of 1,000 anatomic pieces collected over the past 55 years. The pieces were selected in a blind way by the pathologist based on the location and type of the defect exclusively.

## Results

The multiplane rotational acquisition extended the TEE examination by 4 to 6 minutes. An additional time of 15 minutes was necessary for off-line analyses of the 3D images. There were no complications attributed to the TEE procedure. Imaging quality and the corresponding 3D reconstructed structures were considered of good technical quality in all 5 studies. We noticed a high degree of reliability in the 3D TEE reconstructions.

**Figure 1:**
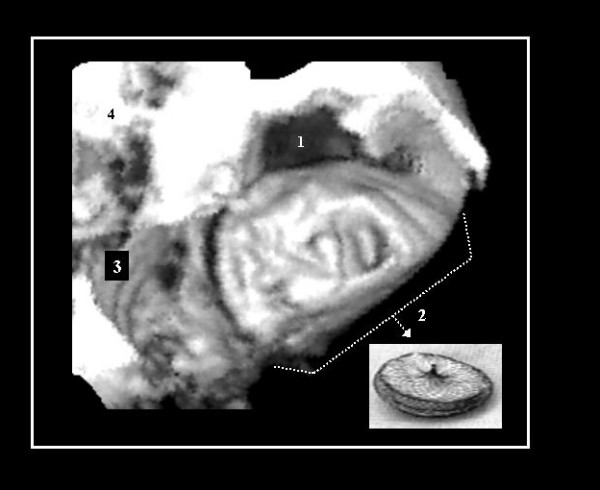
A partially occluded OS ASD with an Amplatzer^® ^device as is seen from a 3D TEE study (left side view). One extension of the defect toward it's superior edge (1) is not covered by the device. This irregularities in the shape may be overlooked in 2D images. 2.- Amplatzer^® ^device as seen in photography and in 3D TEE study; 3.- Mitral ring; 4.- Left ventricle outflow tract.

**Figure 2:**
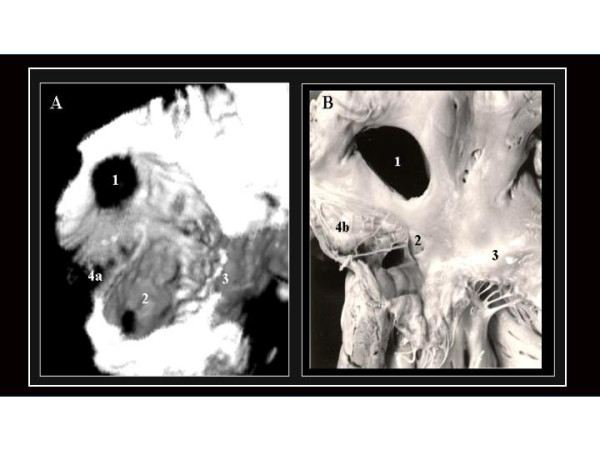
A.- 3D TEE reconstruction illustrating an OS ASD as seen from a right-superior view with removed right atrial free wall. (1). We can observe the orifice of the coronary sinus (2) close to the tricuspid ring (3) forming part of the "Koch triangle". The linear structure between both orifices corresponds to a prominent Eustachian valve (4a). B.- An anatomic specimen that closely resembles the reconstructed 3D image. Note that in this case instead of a prominent Eustachian valve, a Chiari network (4b) is observed.

**Figure 3:**
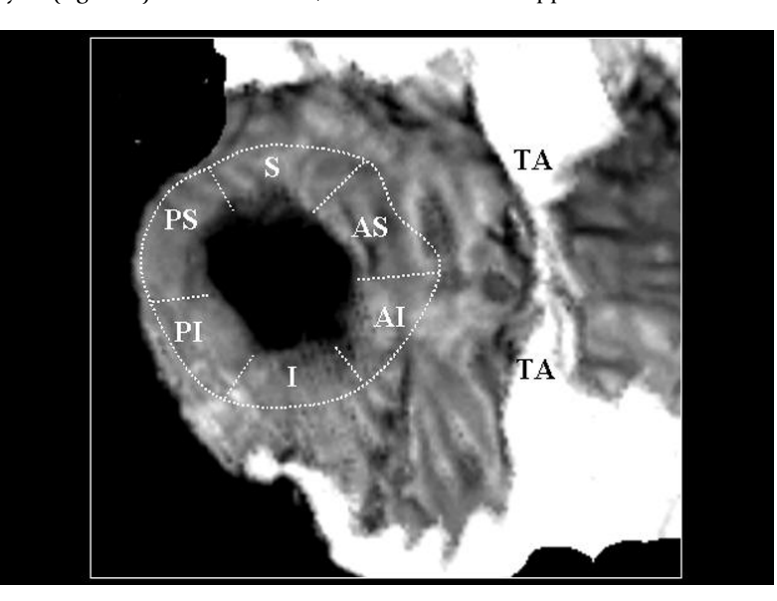
A right-frontal view of the same defect. Locations of 5 sections of atrial septal defect rims proposed by Mathewson and co-workers8 are labeled. S: superior; AS: anterosuperior; PS: posterosuperior; I: inferior; PI: pos teroinferior. SI: superoinferior. TA: Tricuspid annnulus..

**Figure 4:**
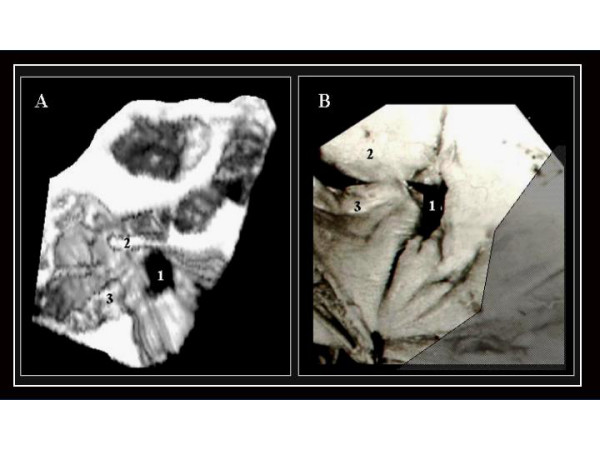
3D TEE reconstruction (A) of the interatrial septum as seen from the left side, illustrating an OP ASD (1), and an analogous anatomic specimen (B). In both images we can observe the relationship between the defect (1) and the mitral valve internal comissure. 2.- Anterior mitral leaflet. 3.-Posterior mitral leaflet.

**Figure 5:**
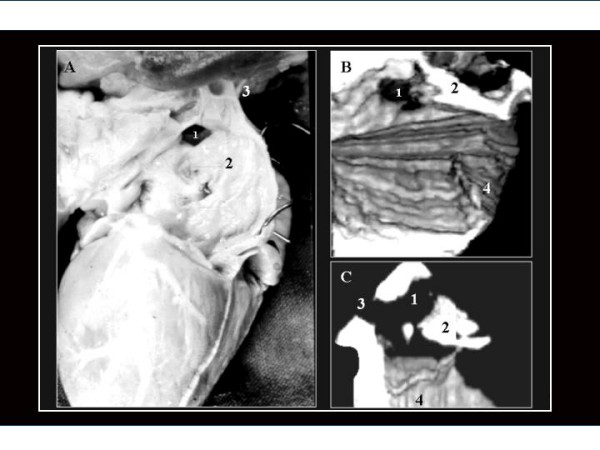
A.- Anatomical specimen of a heart with an VS ASD (1) as is seen from the right side of the interatrial septum (2). B.- 3D TEE image of the interatrial septum in a heart with the same type of ASD as is seen from the right side. C.- A different cut plane obtained form the same 3D-TEE study at the level of the right superior pulmonary vein connection (3) showing its relationship with the defect. 4.-Eustachian valve.

**Figure 6:**
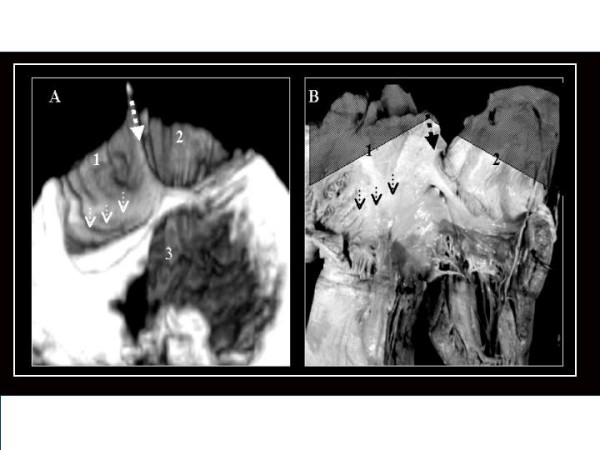
Four chamber view of a heart with CA and tricuspid atresia as seen in a 3D TEE study (A) and an analogous anatomic specimen (B). 1 and 2 indicate the two portions of the CA separated by a small  prominence in its posterior wall (discontinuous arrow). Arrow heads indicate the absence of right AV connection. 3.- A large ventricular septal defect.

**Figure 7:**
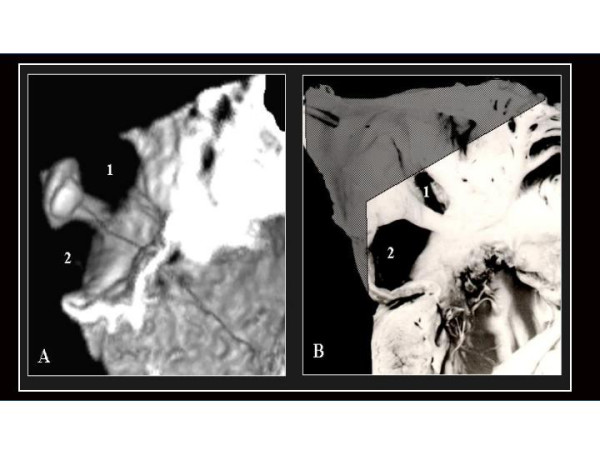
A.- 3D-TEE reconstruction of the interatrial septum as seen from the right side illustrating an OS ASD (1) with a dilated coronary sinus orifice (2) secondary to anomalous pulmonary venous connection. Between both orifices a band corresponding to interatrial septum is observed. B.- An anatomic specimen with the same abnormalities that closely resembles the reconstructed 3D image.

In the first case (figure 1) 3D TEE showed that the shunt was secondary to a residual orifice due to an extension of the defect not well evaluated initially with 2D TEE study. In the second case was possible to observe spatial relationships between ASD, coronary sinus orifice and a prominent Eustachian valve, forming part of the "Koch triangle", (figure 2). In another view of 3D reconstruction, we could visualize the entire endocardial surface of the defect and the dimensions of the 5 portions of the septal rim (figure 3) as has been proposed by Mathewson and co-workers [8]. In the OP case, the spatial relationship between the defect and the mitral valve internal comissure was successfully displayed (figure 4). In the SV case, the defect was associated with anomalous right upper pulmonary venous drainage and one interesting aspect of the 3D reconstruction was the possibility of visualizing it from different angles (figure 5). 3D reconstruction of the CA allowed to appreciate the absence of right AV connection and could accurately display simultaneously the exact geometry of a large ventricular septal defect (figure 6), that closely resembles the surgeon's vision. In the last case, the lack of atrial septal tissue in the 3D image was easily appreciated as well as the distance between the defect and the venous drainage through a dilated coronary sinus orifice (figure 7).

## Discussion

Some differences were observed between the 2D and 3D TEE images. In the 2D studies variations in size were visualized depending on the imaging plane. In the 3D studies, accurate spatial anatomy could be corrected by selecting the appropriate cut plain independently of its orientation. Another advantage of the 3D technique was the ability to actually visualize the entire endocardial surface of the defect, rendering a closer anatomic appearance of the heart. The morphologic analysis of all anatomic specimens with a similar degree of septal anomaly correlated well with the echocardiographic findings and the descriptions of defect shape were the same by 2D and 3D studies.

In literature, the real-time 3D TTE has been evaluated for the various features of ASD and the atrial septum. It is non-invasive and has been shown to be a accurate diagnostic method to determine ASD location and size [9], so, why is it necessary to acquire a 3D-TEE?. Off-line 3D TEE has demonstrated its capability to define small and complex cardiac structures [7,10]. Although sequential acquisition of multiple triggered 2D image planes is time-consuming, it usually allows to obtain higher spatial resolution images in comparison with real time techniques. This aspect can be important for a better definition of atrial septal anatomy and adjacent structures in planning and performing percutaneous device closure of selected cases of ASDs. Maximal diameter of the defect and dimensions of the septal rims are essential parameters for the selection of optimal cases for device closure and in some cases, 3D-TTE may not provide optimal data. Off-line 3D-TEE might help to improve patient selection and assessment of anatomical details.

It has been demonstrated that 3D-TEE is an useful non-invasive tool for evaluating defects of atrial tabication and other anatomical details [11], and it has a better anatomic correlation with matched anatomic specimens than with the corresponding 2D images alone [10]. With 3D images there was a better spatial appreciation of the surrounding structures providing a more "realistic" conceptualization of the cardiac anatomy, particularly with structures located at different tomographic planes [6,12], and it's dynamic changes during the cardiac cycle [13]. Irregularities in shape of the ASDs can lead to residual shunting that, as in the case of figure 1, may require overlapping septal occlusion devices to treat it [14].

It is important to highlight some pitfalls at the time of the 3D interpretation. One must carefully observe for any mismatch among consecutive slices caused by rotation of the probe, which may lead to anatomy distortion. In addition, because the threshold between the solid and liquid interfaces is manually done, inappropriate adjustment may be an operator-dependent pitfall. This may result in either creation of an artifact or deletion of real anatomic segments. At the time of reconstruction radial artifacts can appear. This kind of problem is typical of the technique and it may be appreciated over the endocardial atrial surface in figure 5.

Considering the wide spectrum of the ASDs and their association with others heart structures it is probable that, in the future, 3D reconstruction of transesophageal images complemented with Doppler analysis will be the technique of choice for studying these patients. Likewise, 3D dynamic echocardiography may represent an excellent method for teaching the pathologic anatomy of diverse complex congenital anomalies, particularly now that patients undergo surgical correction of their malformations at very early ages, limiting the study of anatomic specimens (see additional file 1).

## Conclusion

Dynamic TEE three-dimensional echocardiography enhances the understanding of the anatomy of ASDs and should be an important process in future initiatives for device closures or surgical procedures. Combined with 2D techniques, it is reliable in the preoperative assessment of ASD in adults. This study may be indicated when asymmetrical anatomy is suspected by conventional echocardiography evaluation and may not be necessary if there are unmistakable anatomical information. The question of whether the additional morphologic details obtained by this technique has any significant impact on treatment options of individual patients or not, and the role of novel real time transesophageal transducers, must be investigated.

## Authors' contributions

R FJ carried out image acquisition, conceived of the study, and drafted the manuscript, VB J, VA C, EO J, RC A and MR MA participated in the design and coordination of the study, MC L participated in anatomic specimens obtaining. All authors read and approved the final manuscript.

## Supplementary Material

Additional file 1 Atrial septal defect: Rotational view. This video clip shows high resolution dynamic images obtained by 3D TEE that closely resembles anatomic specimens. Rotating the image we can observe both faces of an ostium secundum atrial septal defect and all intermediate planes. Click here for file
